# Visual Impairment and Low Vision Aids—A Comparison between Children and Adults

**DOI:** 10.3390/jpm13111608

**Published:** 2023-11-14

**Authors:** Madeleine A. Perrault, Gabriele Lauer, Sabine Voss, Berthold Seitz, Barbara Käsmann-Kellner

**Affiliations:** 1 Department of Pediatrics and Neonatology, Saarland University Medical Center, 66421 Homburg, Saar, Germany; 2 Department of Ophthalmology, Saarland University Medical Center, 66421 Homburg, Saar, Germany; gabriele.lauer@uks.eu (G.L.); sabine.voss@uks.eu (S.V.); berthold.seitz@uks.eu (B.S.); barbara.kaesmann-kellner@uks.eu (B.K.-K.)

**Keywords:** low vision, visual impairment, blindness, visual aids, rehabilitation

## Abstract

(1) Background: This study aims to highlight differences in the etiology and fitting of low vision aids in visually impaired children and adolescents in comparison to adults. (2) Methods: A retrospective data collection from visually impaired patients presenting to obtain assistive devices from 1 January 2016 to 30 April 2020 was conducted. A total of 502 patients were included. Inclusion criteria were a minimum age of 4 years and the chart notation of a best-corrected distance visual acuity in the patient record prior to the fitting of magnifying visual aids. (3) Results: Of the 502 patients, 147 (29.3%) were children under the age of 18 years. The most common cause of visual impairment in children was albinism, and in adults, it was age-related macular degeneration (AMD). Children showed better distance visual acuity, with a median of 0.88 logMAR (Logarithm of the Minimum Angle of Resolution) compared to 1.0 in adults (*p* = 0.001). Near visual acuity was also significantly better, with a median of 0.54 logMAR in children compared to 0.9 in adults (*p* < 0.001). Near and distance visual acuity were significantly improved by fitting magnifying visual aids (*p* < 0.001). After fitting, near visual acuity averaged 0.3 logMAR, and distance visual acuity, 0.7. The most commonly prescribed aids were optical vision aids, which 68.5% of the patients received; 43.8% received electronic aids. In children, optical aids were more frequently prescribed, and in adults, electronic and acoustic aids (*p* < 0.001). (4) Conclusion: Visually impaired patients can regain the ability to read and improve distance vision by using individually adapted and tested magnifying vision aids, often with optical aids alone. Differences between children and adults could be discovered in the etiology and severity of visual impairment, as well as in the provision type of low vision aids.

## 1. Introduction

According to the World Health Organization (WHO), visual impairment can be classified by the best-corrected visual acuity in the better seeing eye. Mild vision impairment is defined as a visual acuity worse than 0.3 logMAR, moderate vision impairment as worse than 0.5 logMAR, severe vision impairment as worse than 1.0 logMAR, and blindness as worse than 1.3 logMAR [1].

Worldwide, approximately 252 million people are affected by some degree of vision impairment, of which 36 million are blind as defined by the WHO. Vision impairment does not only affect adults but also affects children and adolescents. According to projections, there were around 19 million visually impaired and blind people worldwide under 15 years of age in the year 2015 [2]. However, due to demographic change and the continuous growth of our population, the prevalence of moderate to severe vision impairment and blindness is expected to increase in the following years. The underlying causes of visual impairment in all age groups and worldwide are manifold and include inborn and acquired visual impairments, and can be divided into treatable conditions such as refractive errors, cataract, and age-related macular degeneration (AMD) and non-treatable conditions such as glaucoma and diabetic retinopathy (DR) [3]. In children, hereditary diseases such as albinism and syndromic diseases in the context of multiple disabilities have been described as the main causes of visual impairment in industrialized countries, whereas (congenital) cataract is the most prevalent reason for childhood blindness in developing countries [2,4].

Due to impaired vision, everyday life is greatly affected in many areas. Both reading ability and distance vision can be impaired so that the recognition of faces and facial expressions; the reading of small print, newspapers, and forms; and the ability to drive can be impaired or may be impossible. These limitations in activities of daily life (ADL) often reduce the quality of life for visually impaired people [5,6]. Children, in whom vision plays a major role in the development of motor, social, and cognitive skills, can suffer from the consequences of the developmental disorder for the rest of their lives. The early provision of low vision aids is therefore useful, in addition to the necessary supportive care (e.g., early visual intervention), in order to achieve the best possible integration into the social environment and an improvement in the quality of life [2,7,8].

The rehabilitation of visually impaired patients covers a wide range of possible methods. Magnifying vision aids are available specifically to improve reading ability. These include, first and foremost, optical aids such as glasses and magnifying glasses, as well as electronic aids such as screen readers, tablet readers, text-to-speech devices, and, recently, also smartphones and tablets. So-called monoculars are also part of the magnifying visual aids category but are used for distance magnification. Other practical steps include the good illumination of rooms, training of eccentric vision, and paying attention to higher contrast, as well as mobility training, using long canes and guide dogs, and further training in practical life skills [5,9]. Several studies have already shown that the provision of assistive devices and rehabilitative services can improve the reading ability and general independence of patients. In addition, the provision of assistive devices has positive effects on the assessment of the quality of life, especially as patients tend to improve social relationships. Further benefits are better mobility and fewer falls in the elderly, as well as decreases in depressive symptoms in affected patients [2,5].

This study focused on the collection and analysis of retrospectively acquired data on visually impaired patients who presented themselves in the “KiOLoN” (Pediatric Ophthalmology, Orthoptics, Low Vision, and Neuro-Ophthalmology) section of the Department of Ophthalmology at the Saarland University Medical Center, Homburg/Saar, Germany. There, the testing and fitting of magnifying vision aids were performed on adults and pediatric patients. The aim was to assess both the current situation of the patients as well as the current treatment standards of low vision therapy. A special focus was the identification of differences in the etiology of visual impairment and the provision of aids for children and adolescents compared to adult patients, with the assumption that children have a higher capacity to compensate their impairment and are thus able to cope better with the adaption of visual aids. An additional interest, especially concerning the elderly patients with acquired low vision, was to find differences in visual aid fitting in spite of having the same underlying disease (i.e., fitting of devices according to person, not disease).

## 2. Materials and Methods

The present study involved a retrospective data collection. Included in the study were patients who presented themselves at the “KiOLoN” section of the Department of Ophthalmology at Saarland University Medical Center, Homburg/Saar in the period from 1 January 2016 (date of introduction of electronic patient records) to 30 April 2020 and had low vision forms in their patient records. In order to receive low vision forms, the patients had to have been seen during the specialized low vision consultation hours. To capture consultative provision of low vision aids, patient records from this time period with diagnosis codes E70.3 “albinism”, Q13.1 “absence of iris (congenital)”, H47.2 “optic atrophy”, H53.5 “achromatopsia”, and H35.5 “hereditary retinal dystrophy” as the main diagnosis were manually reviewed for low vision services and prescription of assistive devices and included in the study, if present. Inclusion criteria were a minimum age of four years at presentation, for better comparability of visual acuity due to the usage of different tests (especially in infants younger than four years of age), and a best-corrected distance visual acuity available in the patient record as baseline before fitting of magnifying vision aids. Patient data previously digitally recorded using Fidus^®^ software (www.fidus.de (accessed on 2 October 2023), Arztservice Wente GmbH, Frankfurter Landstraße 117, 64291 Darmstadt, Germany) were collected in an SPSS database (IBM^®^ SPSS^®^ Statistics, version 26, International Business Machines Corporation (IBM), Armonk, New York, USA) [10,11,12]. A total of 502 patients met the studies’ inclusion criteria and were added into the database.

The information collected in the database was specifically categorized on the following parameters. Said categories were:

- general information and medical history;
- functional findings;
- provision of low vision aids.

General information on medical history included name, gender, age at presentation, and ocular main and secondary diagnoses. Within the category of functional findings, patients’ subjective residual visual function and visual acuity were recorded. For the subjective residual visual function category, patients’ anamnestic data on the ability to perform activities of daily living (ADL) and reading ability were recorded. In particular, the ability to read newspapers and to perform shopping and other duties, as well as daily hygiene and other tasks, such as putting on clothes independently, were given importance in relation to the underlying visual impairment. Children who could not yet read due to age were excluded from this category. Consideration was also given to the respective ages of the children regarding the practicality of the ADL. Visual acuity was recorded for objectifiable classification of the severity of the patients’ vision impairment and assessment of the effectiveness of magnifying vision aids. For this purpose, the visual acuity values of both patient eyes were determined separately as decimal numbers. Another component was the recording of both the binocular near visual acuity before fitting of the magnifying vision aids as well as the near and distance visual acuity with use of the fitted vision aid (cum correctione: c.c.). Newspaper reading with a near visual acuity c.c. of 0.3 logMAR was accredited as a reference to determine magnification requirements. For better comparability, all visual acuities in this publication were recorded in logMAR.

**Figure 1:**
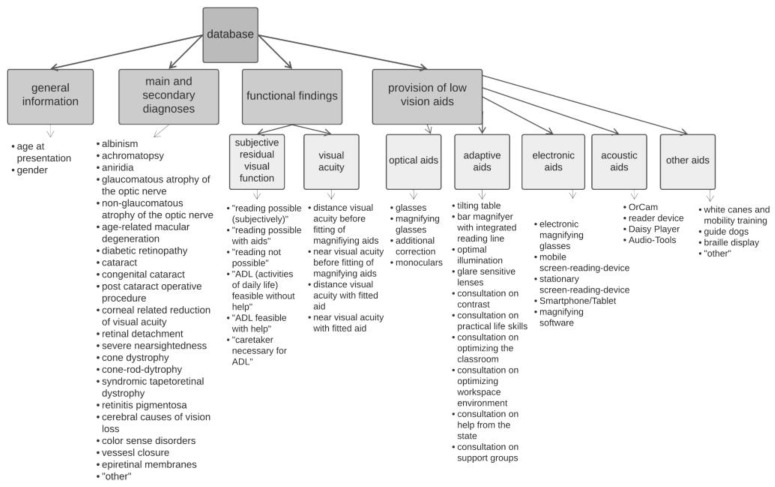
Outline of the parameters recorded in the database.

A central component of the database was the recording of the provision of aids to visually impaired patients. The subdivision was initially conducted into the categories “optical aids”, “electronic aids”, “adaptive aids”, “acoustic aids”, and “other aids”. Each of these categories was then further divided according to different possible aids. Figure 1 shows an overview of the parameters recorded in the database.

Statistical analysis of the data was performed using SPSS software version 26.0 (IBM^®^ SPSS^®^ Statistics, version 26, International Business Machines Corporation (IBM), Armonk, NY, USA). For statistical comparison in independent variables, the Wilcoxon–Mann–Whitney test was used; when comparing dependent variables, the Wilcoxon signed-rank test was used. To detect correlations between the nominally scaled variables, the chi-square test was used. For all statistical tests used, the significance level was set at 5% (α = 0.05).

This study was conducted under the ethical standards of the ethics committee at the Saarland Medical Association (“Ethik-Kommission der Ärztekammer des Saarlandes”) and was approved by the same committee before the data collection was started (ethical approval code: 114/20).

## 3. Results

### 3.1. General Information and Medical History

A total of 502 patients met the inclusion criteria and were included in the study. Of these, 70.7% were adults between the ages of 18 and 99 years (*n* = 355) and 29.3% were children (*n* = 147) aged between 4 and 17 years. For the analysis of certain parameters, the study population was additionally divided into the following age groups: 4–12 years, 13–17 years, 18–30 years, 31–59 years, and over 60 years of age. The age distribution as part of the description of the study collective can be seen in Table 1. The gender ratio was almost balanced, with 51.6% (*n* = 259) female to 48.4% (*n* = 243) male patients. Looking at the gender distribution in children and adolescents separately from that in the adult population, a shift towards the male gender could be observed in children under 18 years, with 59.9% (*n* = 88), and towards the female gender in adults, with 56.3% (*n* = 200).

**Figure 2:**
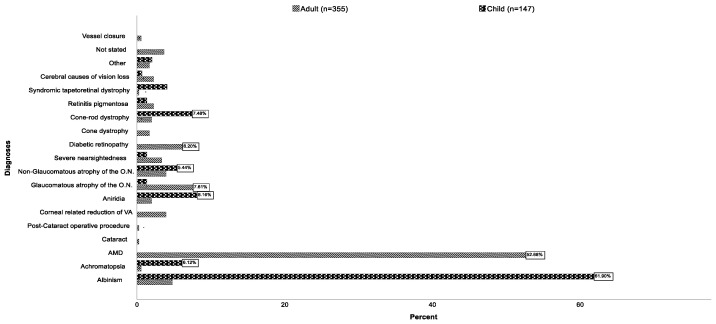
Distribution of the different main diagnoses in children (under 18 years) compared to adults (abbreviations in this diagram: O.N. = optic nerve; VA = visual acuity).

The main diagnosis of each of the patients with the greatest impact on their visual acuity was recorded as the cause of their vision impairment. The most frequent main diagnoses were age-related macular degeneration (AMD), with 37.3% (*n* = 187), and albinism, with 21.5% (*n* = 108). Children showed significantly different causes compared to adults (*p* < 0.001 in the chi-square test). The most frequent main diagnosis in children was albinism, with 61.9% (*n* = 91) and in adults, AMD, with 52.7% (*n* = 187 patients), followed by glaucomatous optic atrophy, with 7.6% (*n* = 27), and diabetic retinopathy, with 6.2% (*n* = 22). Figure 2 shows the differences in the distribution of the various main diagnoses in children compared to adults.

In addition, secondary ocular diagnoses were considered. The most frequent secondary ocular diagnoses were “ post cataract surgery” (*n* = 226, of which *n* = 5 were children), “corneal related vision loss” (*n* = 49, of which *n* = 9 were children), “colour vision defects” (*n* = 47, of which *n* = 26 were children), “cataract” (*n* = 46, of which *n* = 4 were children), and “glaucomatous optic atrophy” (*n* = 46, of which *n* = 10 were children). With increasing age, a significant increase in the frequency of the presence of at least one secondary ocular diagnosis was found (*p* < 0.001 in the chi-square test).

A significant influence on the visual acuity of the patients could be shown for the main diagnoses (*p* = 0.013 in the Wilcoxon–Mann–Whitney test); the presence of secondary diagnoses showed no statistical differences. Patients with aniridia showed the lowest visual acuity, with a mean of 1.1 logMAR, followed by achromatopsia and myopia magna, both with a mean visual acuity of 1.15 logMAR. Patients with cerebral causes of vision loss showed the best visual acuity, with a mean of 0.65 logMAR, followed by Retinitis pigmentosa (mean: 0.7 logMAR) and syndromic tapetoretinal dystrophy (mean: 0.75 logMAR).

### 3.2. Functional Findings

The median visual acuity of both patients’ eyes in the total population was 1.0 logMAR (mean: 0.88, SD ± 0.9) and the binocular near visual acuity was 0.8 logMAR (mean: 0.7, SD ± 0.76). Children showed significantly better distance and near visual acuity even before the aid was fitted (*p* = 0.001 in the Wilcoxon–Mann–Whitney test). The median distance visual acuity in children was 0.9 logMAR (mean: 0.8, SD ± 0.9), compared to 1.0 logMAR in adults (mean: 0.88, SD ± 0.9). The median near visual acuity in children was 0.54 logMAR (mean: 0.6, SD ± 0.76), and in adults, it was 0.9 logMAR (mean: 0.76, SD ± 0.8). Overall, 48% percent of the study population (*n* = 241) could be assigned to the category of moderate vision impairment at presentation according to the WHO classification. Approximately 43% (*n* = 215) already suffered from severe vision impairment or blindness.

Additionally, loss of visual field was included as part of the Low Vision assessment. The concentric restriction of the visual field was the most common cause of loss of visual field, affecting 10% of the study population (*n* = 53). A total of 6% showed central scotoma (*n* = 32). However, 75.5% of the patients (*n* = 375) had no record of loss of visual field. Therefore, no further conclusions could be drawn with regards to the main diagnoses or visual acuity of the patients in this study.

**Figure 3:**
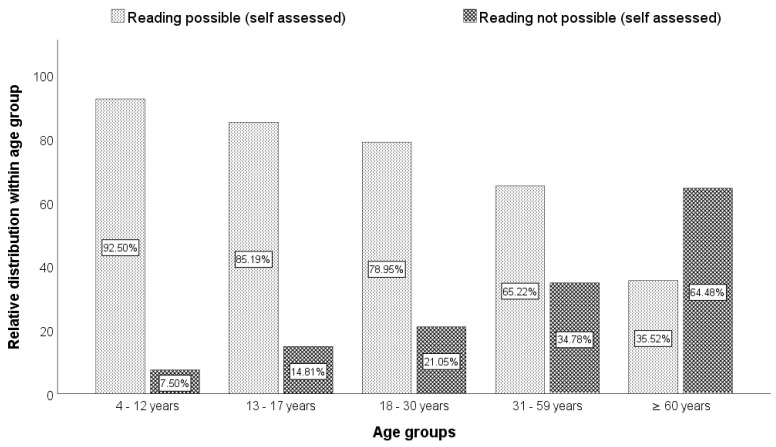
Distribution of the statement “reading subjectively possible” or “reading subjectively no longer possible” in % within the respective age groups.

In addition to the objectifiable visual performance on the basis of visual acuity, anamnestic information on the reading ability and feasibility of activities of daily living (ADL) of the patients was evaluated. A total of 43.8% of the patients (*n* = 220) had lost their ability to read. Adults stated significantly more frequently than children that they could no longer read (*p* < 0.001 in the chi-square test). A total of 58.3% of the adults (*n* = 207) stated that they could no longer read, compared to only 8.8% (*n* = 13) of the children. This could also be shown for the different age groups so that with increasing age, the subjective reading ability was assessed as growing increasingly worse (*p* < 0.001 in the chi-square test). Figure 3 illustrates the reading ability in relation to the age groups of the patients.

Overall, the subjective assessment of visual performance, measured via reading ability, was associated with the patients’ visual acuity so that with decreasing visual acuity, reading ability was also assessed as growing increasingly poor (*p* < 0.001 in the Wilcoxon–Mann–Whitney test). The median visual acuity of patients who reported that they could no longer read was 1.1 logMAR (mean: 0.9, SD ± 1.0). For patients who could still read independently, the median visual acuity was 0.7 logMAR (mean: 0.6, SD ± 0.9). Children also rated their visual ability better than adults in terms of the feasibility of ADL. In the population of the over-17-year-olds, the indication of needing help or complete supervision with ADL occurred significantly more often (*p* < 0.001 in the chi-square test). Again, there was a connection to the patients’ visual acuity so that those with poorer visual acuity more often reported needing partial or full assistance with daily living. The median visual acuity for patients with full assistance was 1.3 logMAR (mean: 1.1, SD ± 1.0). In comparison, the median visual acuity of patients who could perform ADL independently or needed some assistance was 0.9 logMAR (mean: 0.8, SD ± 0.9).

### 3.3. Provision of Low Vision Aids

**Figure 4:**
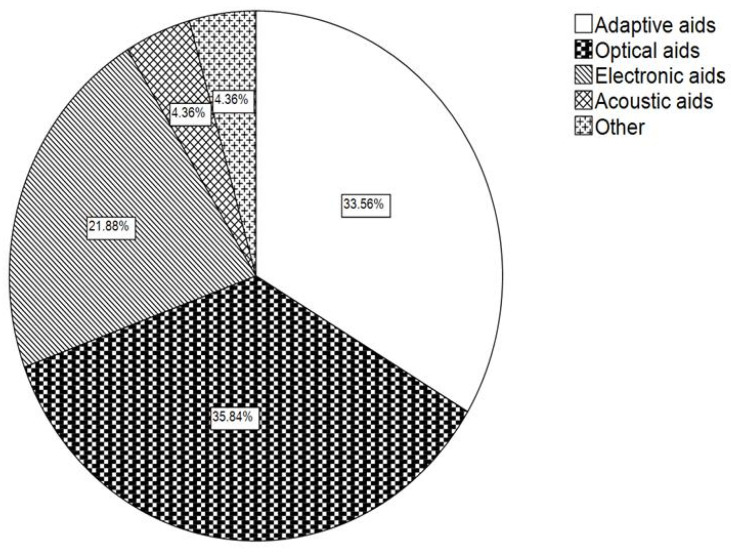
Distribution of the different groups of visual aids in reference to all prescribed aids (*n* = 999).

Finally, the supply of aids to the patients was considered, and the prescribed aids were assigned to the groups named “adaptive”, “optical”, “electronic”, “acoustic”, and “other” aids. Overall, suitable aids could be found for 97.6% (*n* = 490) of the patients. Adaptive aids were prescribed most frequently, constituting 34.5% (*n* = 345) of all aids, followed by optical aids, which constituted 34.4% (*n* = 344). Figure 4 shows the distribution of the different groups of prescribed aids among all fitted low vision aids (*n* = 999 tools).

**Figure 5:**
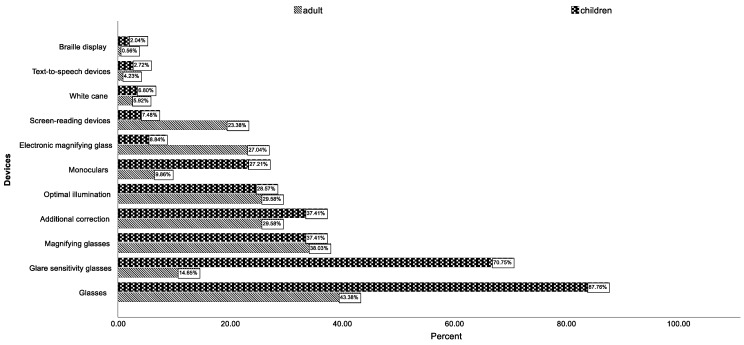
Distribution of the different fitted aids in children and adults in percent format.

Children received optical aids more often than adults (*p* < 0.001 in the chi-square test). A total of 95% (*n* = 114) of the 4–12-year-olds and 88.9% (*n* = 24) of the 13–17-year-olds received at least one visual aid, compared to only 58% of all adults (*n* = 206). In contrast, electronic (*p* = 0.014 in the chi-square test) and acoustic (*p* = 0.002 in the chi-square test) aids were prescribed more frequently for the over-17-year-olds. The group over 60 years of age received electronic aids most frequently, with 50% (*n* = 145) of all patients in this age group having received them. Acoustic aids were also most frequently fitted in the over-60-year-olds in 10% (*n* = 29) of the age group. Figure 5 shows the distribution of the individual aids of the different categories in comparison between children and adults, showing a clear predominance of electronic visual aids in elderly patients.

In addition, a connection between the prescribed devices and the visual acuity of the patients was found. With low visual acuity, electronic, acoustic, and other aids were increasingly prescribed; with better visual acuity, optical and adaptive aids tended to be prescribed. The median distance visual acuity of patients for whom optical aids were not sufficient was 1.3 logMAR (mean: 1.1, SD ± 1.0). Patients who later received an optical aid had a median distance visual acuity of 0.9 logMAR before adjustment (mean: 0.8, SD ± 0.88, *p* < 0.001 as per Wilcoxon–Mann–Whitney test). Patients who had an electronic aid fitted had a median distance visual acuity of 1.1 logMAR (mean: 1.0, SD ± 1.1). The majority of the population (*n* = 382) received more than one device during the fitting of low vision aids. Therefore, no single device could be determined with regards to the best improvement in near visual acuity.

**Figure 6:**
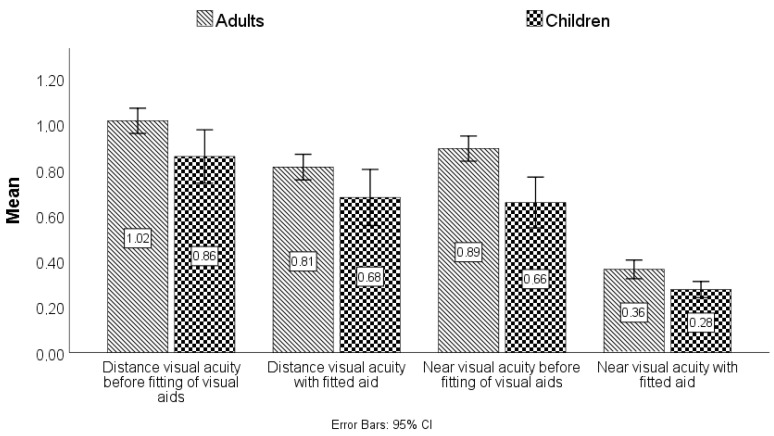
Visual acuity in logMAR (Logarithm of the Minimum Angle of Resolution) for children and adults before and after fitting of visual aids.

Both near and distance visual acuity improved with aid fitting. Distance visual acuity could be improved, on average, by 0.2 logMAR (median: 0.204 logMAR, min.: −1.03 logMAR, max.: 1.05 logMAR). The mean improvement of distance visual acuity in children was 0.18 logMAR, compared to 0.21 logMAR in adults. After adaption, near visual acuity within the total population averaged 0.3 logMAR (SD ± 0.76), which was a statistically significant improvement compared to near visual acuity without aids (*p* < 0.001 in the Wilcoxon signed-rank test). On average, the improvement in near visual acuity equaled 0.51 logMAR (min.: −0.08 logMAR, max.: 2.04 logMAR). There was no statistically significant difference in the use of optical versus electronic aids (*p* = 0.691 in the Wilcoxon–Mann–Whitney test). The mean distance visual acuity with adjusted visual aids was 0.6 logMAR (SD ± 0.7). The greatest possible improvement in distant visual acuity was achieved with monoculars. Loss of contrast sensitivity showed no statistical influence on visual acuity after the fitting of low vision aids. Figure 6 shows the visual acuity values in logMAR for children and adults before and after the fitting of aids to illustrate the effect of using the visual aids.

## 4. Discussion

This study showed that through the provision of individually fitted low vision aids, near and distance visual acuity could be improved, leading to patients regaining the ability to read, which was in line with the findings of other studies [13,14,15,16]. Not only visual acuity but also reading speed can be improved through low vision aids, which Ngyuen et al. (2008)—as well as other studies, such as refs. [16,17]—were able to show. In addition to that, Rohrschneider et al. (2002) [14] and Fröhlich et al. (2006) [13] could demonstrate that in addition to the measurable improvements in visual acuity and reading speed through visual aids, the use, acceptance, and satisfaction with the fitted aids is high amongst visually impaired patients, which again emphasizes the importance of visual aid provision.

As another key message, this study showed that different causes of vision impairment, as well as different ages, should be taken into consideration when fitting visual aids. Despite increasing possibilities in the field of electronic aids, in this study, optical vision aids—magnifying glasses, in particular—were the most frequently fitted low vision aids. In accordance with this, further studies conducted in Germany have shown that optical aids are still the most frequently used and prescribed aids in low vision services [13,17]. They offer both high social acceptance, which can be an important factor for visually impaired patients, as well as a lower cost compared to electronic aids, keeping in mind that health insurance companies might not cover, or only partly cover, the expenses [18]. For adolescents and children, tablets and smartphones, which are usually not yet available on prescription (some insurers provide tablets), offer another form of aid with high social acceptance, and can therefore be used for magnification in many ways [19]. However, conventional electronic aids such as CCTV (closed circuit television) in this study were most commonly fitted in the age group of patients who were 60 years and older, showing that age and social stigmata (e.g., older patients not being able to cope as easily with handling electronic devices as children and adolescents) should not play a role in the fitting of low vision aids. Proper fitting and advice and training in handling a fitted aid, on the other hand, are important parts of low vision services [15]. This statement is further supported through the high amount of adaptive care that patients received in this study population, again emphasizing the need for low vision services.

In addition, visual acuity and the possible progression of the disease also need to be taken into consideration during the fitting of low vision aids as these reflect in the self-assessed ability to read. A significant number of patients (almost 44% of the study population) had already lost their ability to read due to their vision impairments. Children and adolescents, who had significantly better near and distance visual acuity at presentation, rated both their reading ability and the feasibility of performing ADL better than adults due to the better compensation of congenital low vision. In addition, this may have been influenced by many different factors and not merely by visual acuity. Mental and other comorbidities play a major role in the lives of visually impaired patients of all ages [20]. Depression is known to be a frequent comorbidity in vision impairment and could have an impact on the personal assessment of reading ability and ADL, especially with regard to the fact that adults are more frequently affected [21]. Children also show distinctly better compensation of their vision impairment as they are often forced to grow up with it from the beginning due to hereditary diseases whereas adults are often affected later in life, for example, due to AMD. Adults who suffer from late-onset vision impairment and have been used to full visual acuity during their whole lives have severe compensation deficits in comparison to children, including anxiety, insecurity in mobility, and others [8,20,22,23].

Referring to the underlying diseases as the causes for vision impairment, the diagnoses recorded in this study largely coincided with the causes already known in the literature. As in this study, Flaxman et al. (2017) [3] and Nguyen et al. (2008) [17] showed that age-related macular degeneration, glaucomatous optic atrophy, and diabetic retinopathy are the most common conditions in adults with vision impairment. In children, other studies have also shown a wide range of causative diseases that generally include those covered in the study presented here [7,8,24]. The largest proportion of study participants, comprising those over 60 years of age, is therefore defined primarily by the number of patients with age-related macular degeneration. Children present predominantly in the age group of 4–12 years. This is due to the more common hereditary diseases being the causes of vision impairment in children and adolescents, which are therefore also associated with an earlier onset of the disease. The hereditary component also played a role in the gender distribution within this study as, for example, the large proportion of children with X-linked inherited albinism had led to the shift to the male gender in children and adolescents. The higher life expectancy of women in old age, on the other hand, caused the shift to the female sex among adults [25].

As limitations of this study, the bias towards the large number of adults should be stated. This was due to the fact that children in particular have wider access to assistive technology in Germany through early intervention and support measures at kindergarten and school. However, adults could also receive assistive devices, for example, through their general eye-care-practitioners. As this, unfortunately, is not a consistent approach, it leads to leaving out a significant number of visually impaired patients who are not referred to or informed about specialized low vision centers. Additionally, the shift towards children with albinism should be mentioned. The fact that albinism accounted for such a large proportion of diseases in this population was probably due to the fact that the center of medical advisory services for the albinism self-help-group in Germany (“NOAH”: National Organization for Albinism and Hypopigmentation) is located at the Saarland University Medical Center in Homburg/Saar [26]. However, another study conducted in Germany has underlined that hereditary diseases play a great role in the induction of visual impairment in children and has also shown albinism as being one of the leading diseases [27]. Further limitations are due to the retrospective design of this study. Longitudinal studies are needed to further study the use of and satisfaction with low vision aids individually for each patient’s course of disease.

Ultimately, this study showed that near and distance visual acuity could be improved through visual aids, perhaps merely through optical aids such as magnifying glasses alone. With regards to the findings regarding the different etiologies, severity of vision impairment, and differences between children and adolescents compared to adults, it should be acknowledged that the fitting of low vision aids remains a personalized fitting method and should be a source of help and improvement in an individual’s daily life and integration into society. The individual aim should be to have as much magnification as necessary but as little as possible to minimize distortion effects.

Due to demographic change, vision impairment in the elderly is expected to increase in the following years [28]. Therefore, the provision of low vision aids will continue to be a relevant topic in medical care. In view of the progress made with regard to electronic visual aids in particular, the field will most likely remain in flux, and the regular evaluation of aids individually tailored to the patient will be necessary. Adults with a significantly worse visual acuity in comparison to children may benefit more from electronic and acoustic aids with better magnification options. Optical aids may be suited better for younger children as the need for magnification is not as crucial due to the capacity of full accommodation combined with the ability to minimize distance and the fact that the social acceptance of optical visual aids is high. In adolescents, however, peer pressure and the wish to not be different from age mates often lead to the use of smartphones as magnifying aids.

Looking towards the future of low vision services, the progressed stadium of moderate to severe vision impairment, and even blindness at first presentation, need to be taken into consideration. Lam et al. (2013) [29] and Jose et al. (2016) [30] focused on finding out the cause as to why patients, even in industrialized countries, are presenting so late in their course of disease for the specialized fitting of visual aids. Amongst those causes is, mainly, a lack of education on the possibilities of low vision aids on both sides—both patients as well as general eye-care-practitioners. As this study population also mostly presented with progressed vision impairment, educating patients on their diseases and options of rehabilitation is a necessary step in caring for visually impaired patients [31].

Further research might be conducted both in the field of the domestic use of visual aids as well as in keeping up with patterns in the fitting of low vision aids in the course of advancing technical developments. Larger cohorts and multicenter studies could help in gaining a global view of low vision aid provision, as well as in implementing and making use of data collected in registers for the blind and visually impaired.

## Figures and Tables

**Table 1:** Age distribution within the study population.

	Total Sum of Participants in the Respective Age Group	Median Age at Presentation
Overall population	*n* = 502 (100%)	71 years (mean: 54.7 years, SD ± 33.5 years, range: 4–99 years)
4–12 years of age	*n* = 120 (23.9%)	7 years
13–17 years of age	*n* = 27 (5.4%)	14 years
18–30 years of age	*n* = 19 (3.8%)	19 years
31–59 years of age	*n* = 47 (9.3%)	47 years
>60 years of age	*n* = 289 (57.6%)	82 years

## Data Availability

Data sharing is not applicable to this article. The data presented in this study are available on request from the corresponding author. The data are not publicly available due to the privacy of the included patients.

## References

[1] World Health Organization (2019). World Report on Vision.

[2] Elsman E.B.M., Al Baaj M., van Rens G.H.M.B., Sijbrandi W., van den Broek E.G.C., van der Aa H.P.A., Schakel W., Heymans M.W., de Vries R., Vervloed M.P.J. (2019). Interventions to improve functioning, participation, and quality of life in children with visual impairment: A systematic Review. Surv. Ophthalmol..

[3] Flaxman S.R., Bourne R.R.A., Resnikoff S., Ackland P., Braithwaite T., Cicinelli M.V., Das A., Jonas J.B., Keeffe J., Kempen J. (2017). Global causes of blindness and distance vision impairment 1990–2020: A systematic review and meta-analysis. Lancet Glob. Health.

[4] Käsmann-Kellner B., Hille K., Pfau B., Ruprecht K.W. (1998). Eye and general illnesses in the public school for blind and visually handicapped students in Saarland. Developments in the last 20 years. Ophthalmologe.

[5] Shah P., Schwartz S.G., Gartner S., Scott I.U., Flynn H.W. (2018). Low vision services: A practical guide for the clinician. Ther. Adv. Ophthalmol..

[6] Riazi A., Gharebaghi R., Heidary F. (2022). Seven-Year experience in a low vision rehabilitation clinic at a tertiary referral center. Med. Hypothesis Discov. Innov. Optom..

[7] Käsmann-Kellner B., Seitz B. (2021). MDVI patients—Multiply disabled visually impaired: On the situation of the child, parents and ophthalmologist with MDVI children. Ophthalmologe.

[8] Dalal D.M., Shah D. (2022). Low vision in children: Adaptation versus prescribing devices. Acta Sci. Ophthalmol..

[9] Trauzettel-Klosinski S. (2018). Current possibilities of visual rehabilitation. Ophthalmologe.

[10] Maamri A., Fries F.N., Spira-Eppig C., Eppig T., Seitz B. (2022). Employee survey after introduction of the FIDUS electronic patient file at the Saarland University Eye Hospital. Ophthalmologe.

[11] Spira-Eppig C., Eppig T., Bischof M., Schießl G., Milioti G., Käsmann-Kellner B., Carstensen H., Schick B., Seitz B. (2018). Per aspera ad astra: Implementation of electronic patient records in a university eye hospital: Experience with FIDUS in the Clinic for Ophthalmology at the Saarland University Medical Center UKS. Ophthalmologe.

[12] Spira-Eppig C., Eppig T., Bischof M., Schießl G., Milioti G., Käsmann-Kellner B., Carstensen H., Schick B., Seitz B. (2019). Work in progress: Adaptation of electronic medical records to the requirements of a university eye clinic: Individual extensions of the software “FIDUS” at the Department of Ophthalmology of the Saarland University Medical Center UKS. Ophthalmologe.

[13] Fröhlich S.J., Lackerbauer C.A. (2006). Quality control in rehabilitation of patients with visual impairment: Evaluation of use and benefits of optic and electronic devices. Ophthalmologe.

[14] Rohrschneider K., Kiel R., Pavlovska V., Blankenagel A. (2002). Satisfaction with low vision aids. Klin. Monbl. Augenheilkd..

[15] Kloevekorn-Fischer U., Kloevekorn-Norgall K., Duncker G., Grünauer-Kloevekorn C. (2009). Results of low-vision rehabilitation in vision impaired patients. Klin. Monbl. Augenheilkd..

[16] Barker L., Thomas R., Rubin G., Dahlmann-Noor A. (2015). Optical reading aids for children and young people with low vision. Cochrane Database Syst Rev..

[17] Nguyen N.X., Weismann M., Trauzettel-Klosinski S. (2008). Spectrum of ophthalmologic and social rehabilitation at the Tübinger Low-Vision Clinic: A retrospective analysis for 1999–2005. Ophthalmologe.

[18] Pollard T.L., Simpson J.A., Lamoureux E.L., Keeffe J.E. (2003). Barriers to accessing low vision services. Ophthalmic Physiol. Opt..

[19] Martiniello N., Eisenbarth W., Lehane C., Johnson A., Wittich W. (2019). Exploring the use of smartphones and tablets among people with visual impairments: Are mainstream devices replacing the use of traditional visual aids?. Assist. Technol..

[20] Court H., McLean G., Guthrie B., Mercer S.W., Smith D.J. (2014). Visual impairment is associated with physical and mental comorbidities in older adults: A cross-sectional study. BMC Med..

[21] Parravano M., Petri D., Maurutto E., Lucenteforte E., Menchini F., Lanzetta P., Varano M., Van Nispen R.M.A., Virgili G. (2021). Association between visual impairment and depression in patients attending eye clinics: A meta-analysis. JAMA Ophthalmol..

[22] Swenor B.K., Muñoz B., West S.K. (2014). A longitudinal study of the association between visual impairment and mobility performance in older adults: The salisbury eye evaluation study. Am. J. Epidemiol..

[23] Zheng D.D., Swenor B.K., Christ S.L., West S.K., Lam B.L., Lee D.J. (2018). Longitudinal associations between visual impairment and cognitive functioning: The salisbury eye evaluation study. JAMA Ophthalmol..

[24] Haugen O.H., Bredrup C., Rødahl E. (2016). Visual impairment in children and adolescents in Norway. Tidsskr. Den Nor. Laegeforening.

[25] Kolip P., Lange C., Finne E. (2019). Gender equality and the gender gap in life expectancy in Germany. Bundesgesundheitsblatt Gesundheitsforschung Gesundheitsschutz.

[26] Käsmann-Kellner B., Seitz B. (2007). Phenotype of the visual system in oculocutaneous and ocular albinism. Ophthalmologe.

[27] Altpeter E.K., Nguyen N.X. (2017). Ophthalmological rehabilitation of visually impaired children. Ophthalmologe.

[28] Bourne R.R.A., Steinmetz J.D., Flaxman S., Briant P.S., Taylor H.R., Resnikoff S., Casson R.J., Abdoli A., Abu-Gharbieh E., Afshin A. (2021). Trends in prevalence of blindness and distance and near vision impairment over 30 years: An analysis for the global burden of disease study. Lancet Glob. Health.

[29] Lam N., Leat S.J. (2013). Barriers to accessing low-vision care: The patient’s perspective. Can. J. Ophthalmol..

[30] Jose J., Thomas J., Bhakat P., Krithica S. (2016). Awareness, knowledge, and barriers to low vision services among eye care practitioners. Oman J. Ophthalmol..

[31] Wiedemann P. (2022). LOVE your eyes—World Sight Day 2022. Int. J. Ophthalmol..

